# The increasing role of electronic media in headache

**DOI:** 10.1186/s12883-023-03196-5

**Published:** 2023-05-17

**Authors:** Thien Phu Do, Anna P. Andreou, Arao Belitardo de Oliveira, Robert E. Shapiro, Christian Lampl, Faisal Mohammad Amin

**Affiliations:** 1 Department of Neurology, Danish Headache Center, Copenhagen University Hospital - Rigshospitalet, Copenhagen, Denmark; 2 Danish Knowledge Center on Headache Disorders, Glostrup, Denmark; 3 Department of Clinical Medicine, Faculty of Health and Medical Sciences, University of Copenhagen, Copenhagen, Denmark; 4 Headache Centre, Guy’s and St Thomas’ NHS Foundation Trust, London, UK; 5 Headache Research, Wolfson CARD, Institute of Psychiatry, Psychology & Neuroscience, London, UK; 6 Center for Clinical and Epidemiological Research, Hospital Universitário, Universidade de São Paulo – USP, São Paulo, Brazil; 7 Department of Neurological Sciences, Larner College of Medicine, University of Vermont, Burlington, VT 05401-3456 USA; 8 Department of Neurology, Headache Medical Center, Koventhopsital Barmherzige Brüder Linz, Linz, Austria; 9 Department of Geriatric Medicine, Ordensklinikum Linz, Linz, Austria; 10 Department of Brain and Spinal Cord Injury, Copenhagen University Hospital - Rigshospitalet, Copenhagen, Denmark

**Keywords:** Social media, Twitter, Google, Facebook, YouTube, Stigma, Migraine, Headache, Altmetric, Infodemic

## Abstract

Most individuals with access to the internet use social media platforms. These platforms represent an excellent opportunity to disseminate knowledge about management and treatment to the benefit of patients. The International Headache Society, The European Headache Federation, and The American Headache Society have electronic media committees to promote and highlight the organizations’ expertise and disseminate research findings. A growing mistrust in science has made dealing with infodemics (i.e., sudden access to excessive unvetted information) an increasing part of clinical management. An increasing role of these committees will be to address this challenge. As an example, recent studies have demonstrated that the most popular online content on migraine management is not evidence-based and is disseminated by for-profit organizations. As healthcare professionals and members of professional headache organizations, we are obliged to prioritize knowledge dissemination. A progressive social media strategy is associated not only with increased online visibility and outreach, but also with a higher scientific interest. To identify gaps and barriers, future research should assess the range of available information on headache disorders in electronic media, characterize direct and indirect consequences on clinical management, and recognize best practice and strategies to improve our communication on internet-based communication platforms. In turn, these efforts will reduce the burden of headache disorders by facilitating improved education of both patients and providers.

## Introduction

Modern electronic media, hereunder social media, include internet-based communication platforms that allow for direct and indirect information exchange between parties. The most dominant social media sites include Facebook and YouTube with more than two-thirds of US adults using either on a regular basis [1]. As internet and social media usage continues to grow, these platforms represent an excellent opportunity to disseminate knowledge about management and treatment to the benefit of our patients.

## Physician-patient perspective

The electronic media committees of the professional organizations in headache – The International Headache Society, The European Headache Federation, and The American Headache Society – have used social media to promote and highlight their expertise and disseminate research findings to both patients and healthcare providers. This dissemination predominantly occurs through their websites and social media profiles. Social media have changed how we, as healthcare providers, project our image and how patients view and interact with us. For patients and their families, these direct communication channels offer easy access to medical information from experts. Even so, caution must be exercised. These platforms also provide an opportunity for profit-seeking individuals to take advantage of personal circumstances. The information obtained through these sources may not be accurate. The most popular content on migraine management on Google and YouTube is not evidence-based and is disseminated by for-profit organizations, which may lead to inadequate treatment, possibly harmful interventions, and challenging patient-physician interactions [2, 3]. The most popular migraine-related videos on YouTube have been viewed more than 163 million times, so there is a widespread interest in headache-related content and the potential impact cannot be ignored [2]. Users are broadly interested in either symptoms or management (Table 1) [4]. Similar findings have been reported in other fields [5–7]. Misinformation may also lead to mismanagement for individuals who do not seek out conventional medical care, which is a major concern in several regions. Amongst individuals with headache with the highest disease burden, there is a strikingly low healthcare utilization, even in countries that provide this care for free [8, 9]. Moreover, stereotypical portrayals of people with headache disorders, in particular migraine, may contribute to social stigmatization and lack of validation of headache-attributed disability [10, 11].

**Table 1:** The top 25 related queries to the search term “headache” or “migraine” on Google for the past 5 years

Ranking	Headache	Migraine
1	sinus headache	headache
2	headaches	migraine headache
3	nausea headache	migraine symptoms
4	nausea	excedrin
5	tension headache	excedrin migraine
6	migraine headache	migraines
7	migraine	headaches
8	headache covid	migraine headaches
9	covid headache	ocular migraine
10	pressure headache	migraine pain
11	eye headache	migraine aura
12	neck headache	aura
13	fever headache	migraine medicine
14	get rid of headache	migraine relief
15	back of head headache	migraine medication
16	headache causes	what is migraine
17	how to get rid of headache	migraine treatment
18	bad headache	migraine causes
19	i have a headache	eye migraine
20	headache medicine	get rid of migraine
21	headache sore throat	how to get rid of migraine
22	headache relief	migraine help
23	headache and nausea	migraine medications
24	headache pregnancy	migraine piercing
25	severe headache	icd 10 migraine

Data derives from the Google Trends database and covers the time period October 8, 2016 to October 8, 2021 [4]. Queries are broadly related to either symptoms or management

## Infodemics

Infodemic is a portmanteau of “information” and “epidemic” and refers to an accelerated increase of both accurate and inaccurate information about a topic, such as a disease. A growing mistrust in science has made dealing with infodemics an increasing part of clinical management, especially due to some people’s overreliance on social media as their sole source of healthcare information. Healthcare providers in the digital age have an opportunity to try to influence the role those powerful social media platforms may play in the practice of medicine. Otherwise, we risk that our future patient interactions will be shaped by agendas which may not always be beneficial. A recent example can be drawn from the coronavirus disease (COVID)-19 pandemic. Endorsements of chloroquine and hydroxychloroquine by high-profile figures have affected the interest in, and demand for, these drugs for COVID-19 management although the evidence for the benefits vs. harms of these interventions is very limited [12].

A progressive and proactive strategy from professional organizations is necessary to ensure dissemination of evidence-based diagnosis and management information. Most individuals with headache either have tension-type headache, low-frequency episodic migraine, or both headache disorders, which in many cases can be adequately managed with simple analgesics [13, 14]. Nonsteroidal anti-inflammatory drugs (NSAIDs) are generally accessible regardless of region with abundant evidence supporting their use for cost-effective management of headache, even in low- and middle-income countries [13, 15]. Likewise, triptans and preventive medications for migraine are also available in most regions. However, insufficient awareness and understanding amongst both patients and providers limits access to these medications. In some regions, migraine is even not recognized as a neurobiological disorder, [15] which further increases demand for validated information from sources other than formal healthcare institutions. Furthermore, headache disorders continue to be stigmatized, [10] which represents a significant contributor to underutilization of health services [16].

## Public outreach

Fortunately, most individuals affected by headache disorders do not have rare or complicated presentations that requires specialist services, rather, they often rely on self-treatment and self-education [13, 15]. As healthcare professionals and members of professional headache organizations, we are obliged to prioritize patient education and knowledge dissemination, not only of groundbreaking and pioneering research, but also of fundamental information to patients and providers. Of note, randomized trials have demonstrated that a social media strategy of Twitter promotion is not only associated with increased online visibility and outreach amongst the public, but also with a higher number of citations in scientific journals amongst the scientific community [17, 18].

More than 1 billion individuals are affected by migraine worldwide [15]. Social media are viable access-points to educate this population. To date, these efforts have been impactful but there is room for improvement. An alternative metric for outreach of scientific publications is by online mentions, commonly done through Altmetric, that records number of mentions on social media (e.g., Twitter), online news media, and potential individuals exposed to these mentions (amongst other benchmarks). The Danish Headache Society, a national society that does not yet have an established social media strategy, published their reference program for diagnosis and treatment of headache disorders [19]. This document has less than 10 related tweets on Twitter with an upper bound of ~ 5,000 exposures on social media [20]. In comparison, The American Headache Society prioritizes online outreach with dedicated resources and a social media strategy that includes several platforms. In a comparable timespan, their publication on integrating new migraine treatments into clinical practice had an approximate hundred related tweets on Twitter with an upper bound of ~ 300,000 social media interactions [21, 22]. These numbers can be even higher. A recent open access ten step guide to migraine management endorsed by the European Headache Federation and the European Academy of Neurology [23] has so far achieved an upper bound of more than 3 million interactions [24]. These three guidelines serve as examples of how progressive social media strategies and collaborations across societies can increase outreach. Arguably, there are differences in target population and relevance (i.e., national vs. international societies), but we can and should try to optimize factors that maximize validated information outreach.

## Social media presence and engagement

Content from peers and patients are viewed more often and for a longer duration compared to videos from healthcare providers [2]. This is consistent with the fact that less than one-tenth of the most popular migraine-related videos on YouTube are provided by healthcare professionals [2]. Content-wise, complementary and alternative medicine (CAM) and non-pharmacological approaches are highly valued by health consumers, and this is also reflected within headache disorders even if data on potential gain of these therapies are discordant [2, 3, 15, 25–27]. Multiple reasons may account for these discrepancies, [2] including inadequate physician-patient communication about headache cause and management [28, 29]. Treatment decisions are highly influenced by anecdotal information rather than statistical information [30]. This may provide an explanation for why information provided by peers is more likely accepted by patients, and why content on evidence-based medicine fares worse than individual experiences when it comes to outreach [31]. Furthermore, inclusion of anecdotal information not only has impact on outreach, but also on treatment decision-making processes [32]. The influence of anecdotes is greater when statistical information is presented in prose [30]. Whereas, presenting statistical information in pictographs is a simple measure to increase impact and outreach, thereby reducing the influence of anecdotal reasoning in the social media sphere [30]. Other variables that affect outreach of science content on social media includes humorous content and likeability that are both positively correlated with probability to follow additional content from the creator on social media [33]. Personification of social media profiles belonging to brands is becoming increasingly popular for the same reason [34–36]. However, factual knowledge levels of the recipient affect receptivity to the message, [33] and baseline knowledge of disease and management is a relatively unexplored aspect of individuals with headache. Another perspective is directly engaging with patients’ questions and concerns on social media. As a matter of fact, regulatory agencies suggest therapeutic drug and device manufacturers should screen digital media for putative adverse reactions, and that these reports should be handled similarly to spontaneous reports [37]. While social media platforms are an obvious venue for this purpose, its usage for pharmacovigilance is still in its infancy [38].

## The future is now

As health care providers, we have a collective responsibility to provide clear information to our patients and peers to increase health literacy on headache. The key to dealing with the growing infodemic in headache disorders is not only to generate more knowledge, but also to address the current landscape of available information and disseminate the knowledge we already have on clinical management. We acknowledge The International Headache Society, the European Headache Federation and the American Headache Society have all prioritized running several campaigns on different social media platforms with notable influence, but nonetheless, concerted efforts should be made to develop tools for assessment of impact, identify factors for increased outreach, pinpoint areas for improvement and develop effective strategies to facilitate improved outreach. Future research should assess the range of available information on headache disorders in electronic media, characterize direct and indirect consequences on clinical management, and identify best practice and strategies to improve our communication on internet-based communication platforms. In turn, these efforts will reduce the burden of headache disorders by facilitating improved education of both patients and providers.

## Data Availability

Data sharing is not applicable to this article as no new data were created or analyzed in this study.

## Glossary

COVID: Coronavirus disease
NSAID: Nonsteroidal anti-inflammatory drug
